# Multi-Focus Image Fusion Using Focal Area Extraction in a Large Quantity of Microscopic Images

**DOI:** 10.3390/s21217371

**Published:** 2021-11-05

**Authors:** Jiyoung Lee, Seunghyun Jang, Jungbin Lee, Taehan Kim, Seonghan Kim, Jongbum Seo, Ki Hean Kim, Sejung Yang

**Affiliations:** 1 Department of Biomedical Engineering, College of Software and Digital Healthcare Convergence, Yonsei University, Wonju 26493, Korea; amyjylee95@yonsei.ac.kr (J.L.); jangbi3486@yonsei.ac.kr (S.J.); taehan5479@gmail.com (T.K.); jongbums@yonsei.ac.kr (J.S.); 2 Department of Mechanical Engineering, Pohang University of Science and Technology (POSTECH), Pohang 37673, Korea; jungbinlee@postech.ac.kr (J.L.); seonghan@postech.ac.kr (S.K.)

**Keywords:** image fusion, all-in-focus, depth of field, microscopy

## Abstract

The non-invasive examination of conjunctival goblet cells using a microscope is a novel procedure for the diagnosis of ocular surface diseases. However, it is difficult to generate an all-in-focus image due to the curvature of the eyes and the limited focal depth of the microscope. The microscope acquires multiple images with the axial translation of focus, and the image stack must be processed. Thus, we propose a multi-focus image fusion method to generate an all-in-focus image from multiple microscopic images. First, a bandpass filter is applied to the source images and the focus areas are extracted using Laplacian transformation and thresholding with a morphological operation. Next, a self-adjusting guided filter is applied for the natural connections between local focus images. A window-size-updating method is adopted in the guided filter to reduce the number of parameters. This paper presents a novel algorithm that can operate for a large quantity of images (10 or more) and obtain an all-in-focus image. To quantitatively evaluate the proposed method, two different types of evaluation metrics are used: “full-reference” and “no-reference”. The experimental results demonstrate that this algorithm is robust to noise and capable of preserving local focus information through focal area extraction. Additionally, the proposed method outperforms state-of-the-art approaches in terms of both visual effects and image quality assessments.

## 1. Introduction

Generating all-in-focus images is the process of combining visual information from multiple input images into a single image. The resulting image must contain more accurate, stable, and complete information than the input images, and *N* sets of sub-images from different in-focus images are used to obtain the resulting images, from which all focus areas are fused [1]. This process is accomplished by using multi-focus image fusion (MFIF) techniques and is observed in various fields, including digital photography and medical diagnosis [2].

The non-invasive examination of the conjunctiva using a microscope is a state-of-the-art method to diagnose ocular surface diseases. It is performed by observing and analyzing conjunctival goblet cells, which secrete mucins on the ocular surface to form the mucus layer of the tear film. The mucus layer is important for tear film stability, and many ocular surface diseases are associated with tear film instability. In confocal microscopy, the axial resolution often misses important information in areas when the subject is out of focus, owing to a shallow depth of field (DOF) and small field of view (FOV) up to 500 μm × 500 μm [3,4]. Confocal microscopy includes limitations, such as a relatively slow imaging speed due to the point-scanning method. A wide-field fluorescence microscopy that improves the existing limitations was developed for the non-invasive imaging of conjunctival goblet cells [5]. The new fluorescence microscopy visualizes conjunctival goblet cells in high contrasts via fluorescence labeling with moxifloxacin antibiotic ophthalmic solution. It is specialized for live animal models based on its fast imaging speed and large FOV of 1.6 mm × 1.6 mm, and it has the potential for clinical applications. Nevertheless, a high DOF was required to examine the goblet cells in the tilted conjunctiva. Even the most focused images contain unfocused areas, which implies that they lack important information. To solve this problem, it is necessary to obtain several local focus images with different focus areas, and to then combine them into all-in-focus images.

The MFIF method is mainly divided into the transform-domain and spatial-domain methods [6,7]. Transform-domain methods include image transformation, coefficient fusion, and inverse transformation. Source images are converted into a transform domain, and then the transformed coefficients are merged using a fusion strategy. Li et al. introduced the discrete wavelet transform (DWT) into image fusion [8]. The DWT image fusion method consists of three stages: wavelet transformation, maximum selection, and image fusion. Their method fuses wavelet coefficients using maximum selection based on the absolute values of the maximum values in each window. The values of the wavelet coefficients are then adjusted using a filter, according to the ambient values. However, the DWT does not satisfy shift invariance, which is one of the most important characteristics of image fusion, resulting in incorrect fusion or noise. To solve this problem, an image fusion technique based on the shift-invariant DWT model was proposed, and it achieved better results than the original DWT-based method [9]. In addition to the image fusion methods discussed above, the image fusion techniques using transform-domain methods, such as independent component analyses, discrete cosine transformation, and hybrid image fusion methods combining wavelet transformation and curve transformation were also proposed [10,11].

In spatial-domain methods, source images are fused based on the spatial features of the images. Images are mainly fused using pixel values; such methods are simple to implement and can preserve large amounts of information [12]. Li et al. also introduced a spatial-domain image fusion method based on block division [13]. In this method, the input images are divided into several blocks of a fixed size, and threshold-based fusion rules are applied to obtain the fused blocks. Block-based methods can be enhanced by including threshold processing and block segmentation. Block-based image fusion methods fix the block size that affects the fusion results. To solve this problem, adaptive block segmentation methods with different block sizes can be implemented for each input image. The adaptive block method is a quad-tree, block-based method [14]. This method decomposes input images into a quad-tree structure and then detects the focal areas within each block. Additionally, a region-based image fusion method was developed to increase the flexibility of input images. This method subdivides input images into super pixels using both block-based and region-based characteristics simultaneously [15,16]. The basic goal of image fusion is to improve the visual quality of fused images by dividing the boundaries between focused and defocused areas in the input images. In addition to the transform-domain and spatial-domain methods, various hybrid methods and deep learning methods were proposed [17,18,19].

In this paper, we propose a novel MFIF method that analyzes sequences of up to 20 microscopy input images corresponding to different DOF levels. This method is optimized for the newly developed microscope and can analyze goblet cells through results with high DOF. We solve the problems in both the transform domain and spatial domain and present a method for image fusion based on focus area detection. To evaluate the effectiveness of the proposed method, we conduct the application of our method to camera images and conjunctival goblet cell images.

## 2. Materials and Methods

### 2.1. Proposed Method

**Figure 1:**
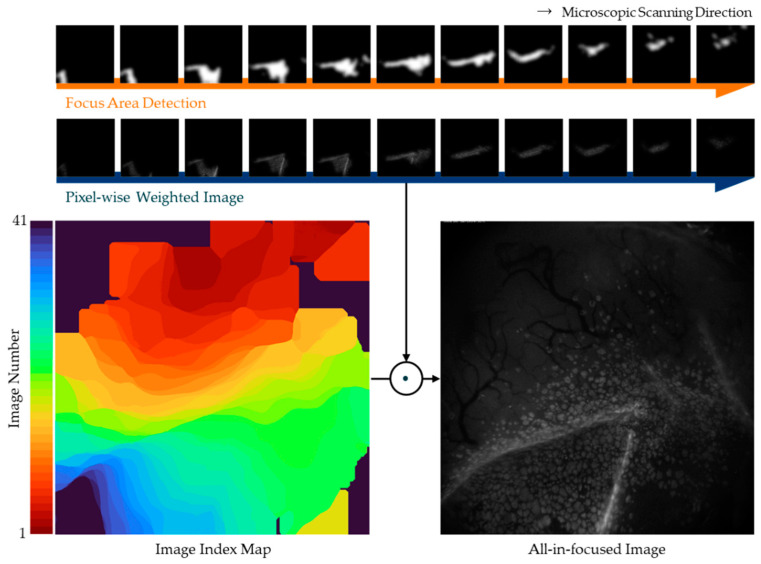
Schematic diagram of the proposed method.

Figure 1 presents a schematic diagram of an image fusion method including focal area extraction. The proposed method is applicable to a large quantity of local-focus images to generate an all-in-focus image. Let *I_n_* be the set of input image sets. First, we adopt a band-pass filter to all filters of the input image sets to enhance the gradient information and edges of the local-focus areas. We then utilize Laplacian filters to enhance the focus areas and thresholding to extract the focus areas, which are denoted as $I_{thn}$. Next, a guided filter is applied, after removing unnecessary areas, by dilating the focus areas. Finally, the focus areas, $I_{gn}$, outputted by the guided filter are combined using the pixel-wise weighted averaging rule and an all-in-focus image is obtained.

### 2.2. Subjects

In this study, moxifloxacin-based, axially swept, wide-field fluorescence microscopy (WFFM) was employed. The objective lens was initially positioned so that the focal plane was at the deepest location of the specimen surface. Then, the focal plane was swept outward by the translation of the objective lens with continuous WFFM imaging. The imaging field of view (FOV) was 1.6 mm × 1.6 mm, the image resolution was 1.3 µm, and imaging speed was 30 frames/s. The WFFM system had a shallow DOF of approximately 30 µm. Typical images had 2048 × 2048 gray scale pixels. Seven 8-week-old SKH1-Hrhr male mice were used for in vivo GC imaging experiment [5].

### 2.3. Focus Area Enhancement Based on the Transform Domain

Local focus images obtained using a microscope require denoising and focus area extraction. A defocus area has a narrower bandwidth than a focus area [20]. Therefore, a focus area has higher frequency information than a defocus area [21,22]. This section presents a method for enhancing focus areas handling the transformation domain.

For domain transformation, we used a Fourier transform to extract high-frequency information and perform denoising simultaneously by applying a band-pass filter. This filter was designed in a Gaussian form, and an appropriate cutoff frequency value was set:

(1) $$F_{n}\left(u,v\right)=fft\left(I_{n}\left(x,y\right)\right),\;n=1\ldots N,$$

(2) $$I_{bpn}\left(x,y\right)=fft^{-1}\left(H_{b}\left(u,v\right)F_{n}\left(u,v\right)\right),\;n=1\ldots N,$$

where $I_{n}$ is an input image with *N* datasets, and fft denotes a Fourier transform; $I_{bpn}$ is a result image with denoising and focus area enhancement performed using the band-pass filter.

### 2.4. Focus Area Detection

After deriving $I_{bpn}$ using a band-pass filter, we applied a Laplacian filter, which is an edge detection method. This filter was employed to compute the second derivative of an image by measuring the rate at which the first derivative changes. This determined whether a change in adjacent pixel values was caused by an edge or continuous progression [23]:

(3) $$I_{ln}\left(x,y\right)=L(I_{bpn}\left(x,y\right),\;M)$$

Here, *L* denotes a Laplacian filter, and $I_{bpn}$ and M are inputs; M is an r×r Laplacian mask, where r must be an odd number. The sum of all the elements in the mask should be zero. Laplacian filters extract edges according to differences in brightness. Because they react strongly to thin lines or points in an image, they are suitable for thresholding [24]:

(4) $$I_{thn}=\{\begin{array}{c} 1,\;\;\;if\;\;\;\;\;\tau_{1}<I_{ln}\left(x,y\right)<\tau_{2}\;\\ 0,\;\;\;otherwise\;\;\;\;\;\;\;\;\;\;\;\;\;\;\;\;\;\;\;\;\;\;\;\; \end{array}$$

Thresholding is the simplest method for segmenting images. Thresholding methods replace each pixel in an image with a black pixel if the pixel intensity is less than a fixed constant. To remove unnecessary areas after thresholding, areas with a small number of remaining pixels are removed. Laplacian filter detects only the edge located at the center of the changing area. Additionally, it is evident that thresholding the filtered image results in a narrower focus area. Therefore, we reconstructed the focal region as a morphological operation [25,26]:

(5) $$I_{dn}\left(x,y\right)=I_{thn}\left(x,y\right)⨁S_{n}$$

**Figure 2:**
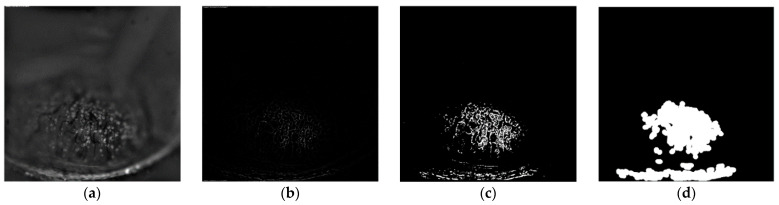
Results of local focus area detection. (**a**) Band-pass-filtered image, (**b**) Laplacian-filtered image, (**c**) thresholded image, and (**d**) dilated image.

In a morphological operation, each pixel in an image is adjusted based on the values of other pixels in its neighborhood. Assume that the structural element for area dilation is defined as $S_{n}$; if the structure element overlaps with a pixel in an input image, then the input image, $I_{thn}$ is expanded. Figure 2 presents the results of the operations discussed above.

### 2.5. Self-Adjusting Guided Filtered Image Fusion

The guided filter method was first proposed by He et al. [27]. A guided filter is a filter that preserves edges, e.g., a bilateral filter. A guided filter kernel is fast, regardless of its size and strength range, and is not impeded by a directional reversal structure. Guided filters were often used in image fusion in previous studies; thus, we optimized the filtering method to fit our algorithm:

(6) $$I_{gni}\left(x,y\right)=a_{k}I_{dni}+b_{k},\forall i\in r_{k}$$

(7) $$r_{k}=1+\left(n-1\right)\times s,\;n=1,2,3\cdots m$$

(8) $$m=\frac{1}{4}\times image\;size$$

A guided filter assumes that an output $I_{gn}$ is a linear transformation of a guidance image $I_{dn}$ in a window centered on a pixel *k*, where $r_{k}$ is the window, and $a_{k}$ and $b_{k}$ are linear correlation coefficients that minimize the squared difference between an output image $I_{gni}$ and input image $I_{mi}$:

(9) $$I_{mi}\left(x,y\right)=I_{n}\left(x,y\right)\times I_{dn}\left(x,y\right)$$

(10) $$E\left(a_{k},b_{k}\right)=\underset{i\in r_{k}}{\sum }\left({\left(a_{k}I_{dni}+b_{k}-I_{mi}\right)}^{2}+\epsilon a_{k}^{2}\right)$$

When the center pixel k changes, the result image $I_{gn}$ also changes. In order to reduce this variation, the result image is determined by averaging the estimates from $a_{k}$ and $b_{k}$.

**Figure 3:**
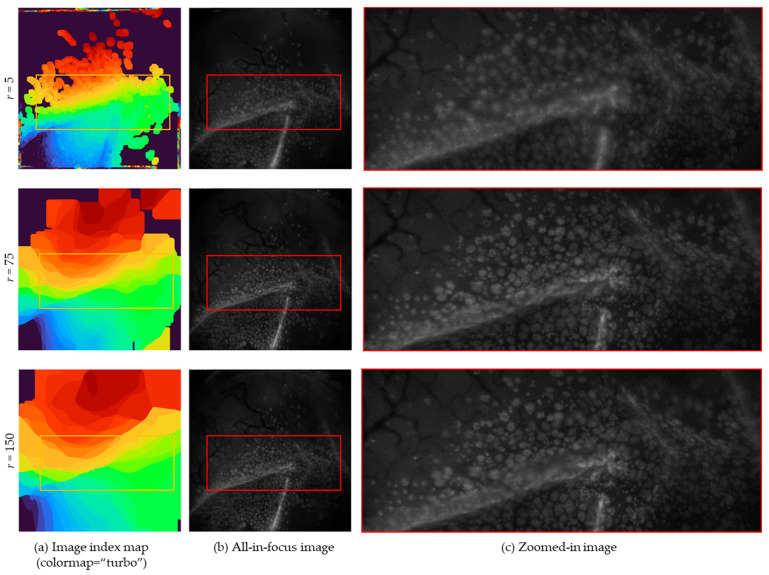
Image index map and processed results for different values of the window size *r*. Each column is separated by a *r*. (**b**) All-in-focus images that are fused based on image index maps in (**a**). The marked areas highlighted by the red box in (**b**) represent the zoomed-in images (**c**). By adjusting *r*, the area affected by the filter is also adjusted. If *r* is 5, as shown in the first column, it did not properly express the boundary features. In the third column, most areas in the image index map are indexed. Since information is extracted from a wide area, there is a disadvantage of obtaining information in an out-of-focus area. As shown in the second column, by choosing an appropriate *r*, a clear fusion result can be obtained without loss of features.

The guided filter was utilized in a sliding window, and filters are applied to the target area according to the size of the window. However, it should be able to weight the image boundaries while preserving a wide area. To select an accurate focus area according to the microscope’s field-of-view area, the window size was automatically adjusted with a self-adjusting guided filter [28]. Therefore, the guided, filtered image, which was affected by window size, was expanded to one-quarter of the size of the entire image, which accelerated the parameter adjustment process. The scale factor s determines the rate of expansion. Thus, we set factor *s* = 2 in the experiment. Figure 3 presents the results for various values of the window size *r*. If the *r* is set to a small value, a gap between the fused areas occurs. On the contrary, if the *r* is set too large, a fusion occurs with unnecessary parts of the image, making it impossible to create a natural all-in-focus image.

After multiplying the original image by the local focus area extraction mask, the focus areas obtained for each image were combined into a single all-in-focus image. Each image had a different focus area; therefore, different image sequence values were included. Additionally, for overlapping focus areas, we used the pixel-wise weighted averaging rule. The pixel-wise weighted averaging rule refers to the method of assigning weights to compensate for the brightness of images during the process of blending between pixels. The final focus area mask produced by the guided filter becomes blurred from the inside to the boundary lines, resulting in smaller pixel values. These pixel values are then regarded as weights. When the source images are fused with respect to the weights, smoothing results are obtained, while maintaining the boundaries between images. The procedure is shown in Algorithm 1.

**Algorithm 1** Multi-focus image fusion algorithm.1: **Input** $I_{N}$: Source images from fluorescence microscopies.2: **Output** F, All-in-focus image.3://Obtain guided filtered focus map of source images4://Obtain output F by selecting the pixels (i,j) from the set of source images, which depends on the calculated weight of the guidance image $I_{gni}$ for the respective pixels.5: **for** i=1: p6:    **for** j=1:q 7:      $I_{arm}\left(k\right)=argsort\left(I_{gni}\left(i,j\right)\right)$8:    //Arrange the calculated weights of the guidance image with respect to the source images.9:        **for** k=1:N//where N is the number of source images to be fused10:          $F\left(i,j\right)+=I_{arm}\left(k\right)\cdot I_{k}\left(i,j\right)$11:      //Obtain output F by sequentially multiplying the source with the maximum weight.12:        **end for**13:    **end for**14:  **end for**

### 2.6. Objective Evaluation Metrics

An objective evaluation of fused images is difficult because there are no standard metrics for evaluating the image fusion process. “Full-reference” condition represents that reference image is secured, and there is a “no-reference” or “blind” condition where reference images are not available, as in many real applications. The image used in the first experiment is “Full-reference” condition, and the dataset used in the second experiment is “blind” condition [29]. Therefore, the following objective assessment metrics were applied according to the conditions.

First of all, there are the “Full-reference” state-only evaluation methods: $Q_{MI}$ is an information-based convergence indicator based on a normalization that overcomes the instability of mutual-information-based indicators. It was proposed by Hossny et al. [30]:

(11) $$Q_{MI}=2\left[\frac{MI\left(A,F\right)}{H\left(A\right)+H\left(F\right)}+\frac{MI\left(B,F\right)}{H\left(B\right)+H\left(F\right)}\right]$$

Here, H(X) is the entropy of the image, and MI(X,Y) is the mutual information value between two images, X and Y.

$Q_{NCIE}$ is an information-based fusion indicator proposed by Wang et al. [31]; $\lambda_{i}$ denotes the eigenvalues of a nonlinear correlation matrix:

(12) $$Q_{NCIE}=1+\overset{3}{\underset{i=1}{\sum }}\frac{\lambda_{i}}{3}log_{256}\frac{\lambda_{i}}{3}$$

$Q_{G}$ is the most-well-known image fusion evaluation metric that measures the degree of gradient information preserved in fused images relative to input images [32]:

(13) QG=∑i=1W∑j=1H[QAF(i,j)ωA(i,j)+QBF(i,j) ωB(i,j)]∑i=1W∑j=1H(ωA(i,j)+ωB(i,j))

Here, the width of the image is *W*, and the height is *H*; $Q^{AF}\left(i,j\right)=Q_{g}^{AF}\left(i,j\right)Q_{\lambda }^{AF}\left(i,j\right),$ and $Q_{g}^{AF}$ and $Q_{\lambda }^{AF}$ are representative of the edge strength and gradient information preserved in the fused image relative to the original image, respectively. The same notation applies to $Q^{BF}$. $\omega^{A}$ and $\omega^{B}$ are the weights of $Q^{AF}$ and $Q^{BF}$, respectively.

$Q_{P}$ is an evaluation metric based on phase congruency. Phase congruency contains prominent feature information from images, such as edge and corner information [33]:

(14) $$Q_{p}={\left(P_{p}\right)}^{\alpha }{\left(P_{M}\right)}^{\beta }{\left(P_{m}\right)}^{\gamma }\;$$

Here, *p*, *M*, and m are the phase congruency, maximum moment, and minimum moment, respectively;$P_{p}$, $P_{M}$, and $P_{m}$ are the maximum correlation coefficients between fused images and input images; and *α*, *β*, and *γ* are the parameters used to adjust the significance of each of the three coefficients, respectively.

$Q_{CB}$ is a method based on the human visual system model. It consists of contrast filtering, local contrast calculation, contrast preservation, and quality guidance methods [34]:

(15) $$Q_{GQM}=\lambda_{A}\left(i,j\right)Q_{AF}\left(i,j\right)+\lambda_{B}\left(i,j\right)Q_{BF}\left(i,j\right)$$

Here, $Q^{AF}$ and $Q^{BF}$ denote the contrast information of the input images preserved in a fused image, and λ denotes the weight value of an input image. $Q_{CB}$ is defined as the mean value of $Q_{GQM}$ as follows:

(16) $$Q_{CB}=\bar{Q_{GQM}}\;$$

Peak signal-to-noise ratio (*PSNR*) and structural similarity index measure (*SSIM*) were used as full-reference quality assessment methods. *PSNR* is an engineering term for the ratio between the maximum possible power of a signal and the power of corrupting noise that affects the fidelity of its representation [35]. *PSNR* is most easily defined via the mean squared error (*MSE*). Given a noise-free m×n image and its noisy approximation, *PSNR* is defined as:

(17) $$PSNR=10log_{10}\left(\frac{MAX_{I}^{2}}{MSE}\right)\;$$

Here, $MAX_{I}$ is the maximum possible pixel value of the image. Because it is measured in logarithmic scale, the unit is dB, and the smaller the loss, the higher the value. For lossless images, the *PSNR* is not defined because the *MSE* is zero.

The *SSIM* is used for measuring the similarity between two images [36]. *SSIM* is a perception-based model that considers image degradation as a perceived change in structural information, while incorporating important perceptual phenomena, including both luminance-masking and contrast-masking terms. The difference with other techniques such as *MSE* or *PSNR* is that these approaches estimate absolute errors. Given an original image and distorted image, *SSIM* is defined as:

(18) $$SSIM\left(A,B\right)=\frac{\left(2\mu_{A}\mu_{B}+C_{1}\right)\left(2\sigma_{AB}+C_{2}\right)}{\left(\mu_{A}^{2}+\mu_{B}^{2}+C_{1}\right)\left(\sigma_{A}^{2}+\sigma_{B}^{2}+C_{2}\right)}$$

Here, $\mu_{A}$ is the average of A, $\sigma_{A}^{2}$ is the variance of A and the same notation applies to $\mu_{B}$ and $\sigma_{B}^{2}$. $\sigma_{AB}$ is the covariance of A and B. $C_{1}$ and $C_{2}$ are two variables to stabilize the division with the weak denominator.

No-reference methods were employed for fused images because reference images are commonly unavailable. One of the most representative no-referenced image quality assessments is BRISQUE, which was introduced by Mittal et al. [37]. BRISQUE is an algorithm that operates on the assumption that if a natural image is distorted, then the statistics of the corresponding image pixels is distorted. A natural image is an initial image captured by a camera that is not processed. Natural images exhibit regular statistical characteristics. The histogram of pixel values takes the form of a Gaussian distribution when processing the MSCN for such an image. For an image quality evaluation, after processing the MSCN, the pixels values were matched with a generalized Gaussian distribution (GGD) to utilize information regarding the pixel distribution as a characteristic feature. The parameters and variance values were compared to the GGD with the most similar forms to evaluate the characteristics of the target image.

Additionally, we defined the NIQE method. This method was also proposed by Mittal et al. [38]. The more similar the output of this method is to a test image, the better the quality of the test image. We also applied preprocessing using MSCN to divide images into patches. We could then derive BRISQUE characteristics within patches and calculate image quality values using mean vectors and covariance metrics.

## 3. Results and Discussion

**Figure 4:**
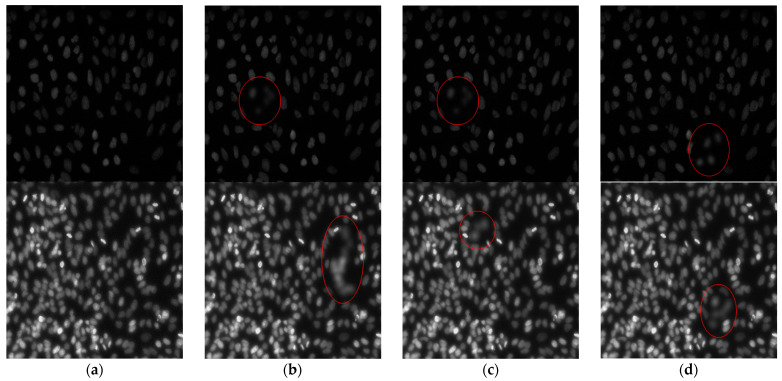
Grayscale image pairs for experiments with random blurring. The top row contains the “Dark cell” image set. The second row contains the “Bright cell” image set. (**a**) Original images and (**b**–**d**) randomly blurred images. From (**b**) to (**d**), we can see that some cells are defocused, and these are marked with a red circle.

In order to verify the proposed method with objective and subjective metrics, we compared its performance with some state-of-the-art methods, such as the discrete wavelet transform (DWT) image fusion, the quad-tree block-based image fusion [8], and Gaussian-filter-based multi-focus image fusion (GFDF) [37]. The first experiment assesses the “Full-reference” condition. We used Kaggle data science bowl 2018 datasets. Since this dataset aims to detect cell nuclei, the prepared data were acquired under various conditions and differed in imaging modalities. We selected two samples which were similar to our microscopic images and named them “Dark cell” and “Bright cell” (Figure 4). For the objective evaluation of the proposed method in this study, Gaussian blurring was applied to the original image to produce three blurred images. The second experiment evaluated the fusion performance by applying the appropriate metrics to the image set with the “blind” condition. The dataset used for the evaluation consisted of conjunctival goblet cell microscopic images taken from a mouse, consisting of 2048 × 2048 grayscale pixels. Each subset contained more than 20 images with different DOFs. All the experiments ware implemented in MATLAB 2019a on an Intel i7-8700 CPU @ 3.20 GHz desktop with 32.00 GB RAM. The proposed method was compared to methods developed in previous works using program codes provided by the original authors [14,39].

**Figure 5:**
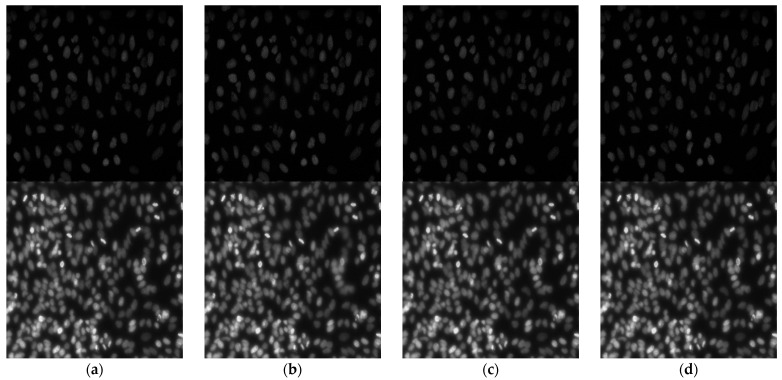
Fused image results generated by different methods. The top row contains the “Dark cell” image set. The bottom row contains the “Bright cell” image set. (**a**) DWT, (**b**) quad-tree, (**c**) GFDF, and the (**d**) proposed method.

**Figure 6:**
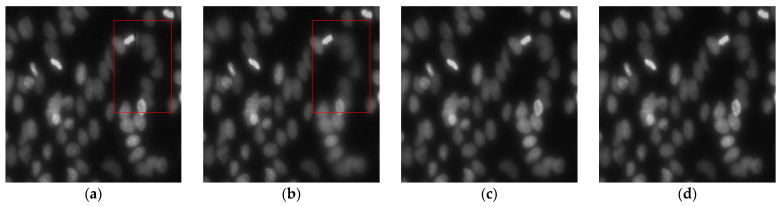
Details of “Bright cell” fused image results generated by different methods. (**a**) DWT, (**b**) quad-tree, (**c**) GFDF, and the (**d**) proposed method.

Figure 5 presents the image fusion results for three images obtained using each MFIF method, and Figure 6 presents the details of the “Bright cell” fusion results. For the DWT and quad-tree methods, the boundaries of the focus areas remain in the resulting images; these areas are marked with a red rectangle in Figure 6. In the GFDF results, the boundaries of the focus areas are not visible, yet some details of the images are missing. The proposed method does not leave boundary lines in the focus areas, and the details of the images are preserved.

The image quality evaluations for the images presented in Figure 6 are listed in Table 1 and Table 2. Comparing the objective metrics reveals that the images fused by the transform-domain methods lose gradient, structure, and edge information. To evaluate the images in the same way, Table 1 and Table 2 are shown as the average of the evaluations of two images among the three input images. The GFDF method utilizes only the absolute difference between two images when detecting the focus area. Therefore, although it shows a high similarity in structure, other image fusion metrics are inferior to those of the other methods when there are more than three input images or overlapping focus areas. Regardless of the number of overlapping focal regions or local focal images, the proposed method is superior in terms of the amount of information loss, results of extracting edges, and quality of the fused results.

**Figure 7:**
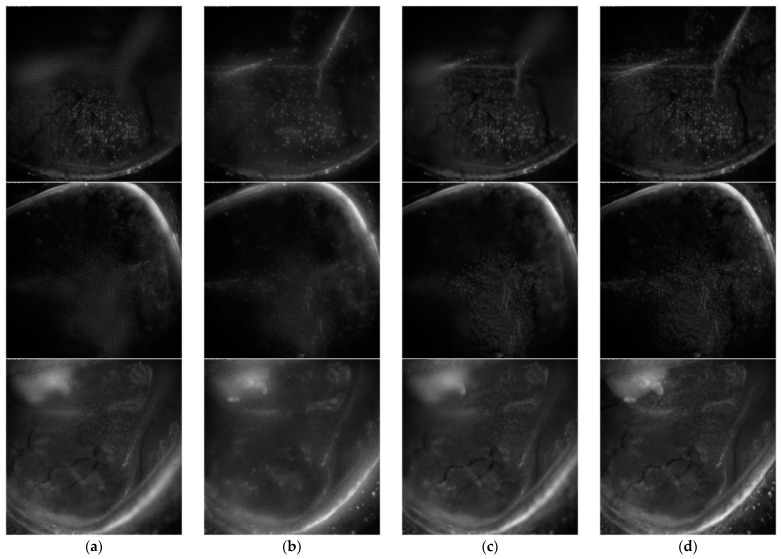
Conjunctival goblet cell, fused image results from different methods. (**a**) DWT, (**b**) quad-tree, (**c**) GFDF, and the (**d**) proposed method.

The second experiment was conducted to evaluate the performance of the proposed method on the “blind” condition image sets. Quad-tree and GFDF algorithms were implemented in two input images. DWT algorithm stated that it was able to fuse 13 images, but the provided code was composed for pair images. Thus, we conducted an experiment in the same conditions as Quad-tree and GFDF. On the contrary, the proposed method is mainly focused on merging more than 20 images at once. Figure 7 presents the image fusion results.

The blind image quality evaluation for the images presented in Figure 7 are listed in Table 3, Table 4 and Table 5. According to Table 3, Table 4 and Table 5, the no-reference image quality assessment indicates the results for each conjunctival goblet cell image. Blind reference-less image spatial quality evaluator (BRISQUE) methods sometimes performed better on GFDFs. However, the naturalness image quality evaluator (NIQE) measurements indicated that the proposed method yielded better results. In the case of the DWT method, one can observe that information loss during image reconstruction is unavoidable. This method suffers from a large amount of information loss in averaging blocks when it is applied to multiple source images. Unlike DWT, the quad-tree method automatically decomposes the window size according to the input image characteristics, but information is not preserved as the number of input images increases. In the case of GFDF, the differences between the images are used to detect focus areas. As the number of source images increases, only the source image information that is fused into the subsequent images is retained as the information from the initially generated focus areas disappears.

From a quantitative perspective, Table 4 indicates that the performance of the proposed method is superior, regardless of the number of source images. The results obtained by the proposed method are more stable and systematic than those of the other fusion methods in terms of the objective evaluation metrics.

## 4. Conclusions

In this work, we presented a multi-focus image fusion method applied to a large quantity of conjunctival microscopic images. Wide-field fluorescence microscopy acquired multiple images with the axial translation of focus, and the large quantity of images transformed into single all-in-focus images through multi-focus image fusion. The proposed method is highly effective in that it performs fusion without being affected by the size and noise of the input image and the number of source images. The proposed method uses the high-frequency characteristics of the focal area to determine the area with a Laplacian filter. Nevertheless, the focus region is detected using the Laplacian filter, and there may be some undetectable parts due to ambiguous boundaries. In addition, the Laplacian filter captures the center of the focus region; we used a morphological operation to compensate for this. The proposed method works on the basis of fixed structural elements, where it is difficult to completely reconstruct the desired area.

However, the experiment was carried out in order to image a live animal model, and the proposed method showed several advantages over previous MFIF methods. First, it prevented visible artifacts such as block shapes and blurring. Additionally, regardless of the number of source images, it was confirmed that an image could be fused using just one iteration and that the proposed method was robust to images with noise. Because differences between microscopic images and general images appear when defining thresholds following a Laplacian transformation, it would be useful to investigate how to select the appropriate thresholds according to the target images. Additionally, developing a better method for the focus area detection is worth additional consideration.

Image fusion techniques are commonly applied in various fields, such as digital photography and medical diagnosis. In particular, it is important that optical microscopic image fusion be performed without losing information. It is expected that both experts and non-experts will be able to fuse images easily using the proposed algorithm.

## Figures and Tables

**Table 1:** Quantitative results for evaluation metrics on the “Dark cell” image with four other methods.

	QMI	QNCIE	QG	QP	QCB	PSNR	SSIM
DWT	1.8534	0.8322	0.9513	0.9363	0.9558	47.3966	0.9851
Quad-tree	1.7286	0.8298	0.9346	0.9064	0.9318	45.5006	0.9811
GFDF	1.4601	0.8242	0.8040	0.8489	0.8436	**47.7980**	**0.9868**
Ours	**1.8** **796**	**0.8327**	**0.95** **19**	0.9354	**0.9** **726**	47.4428	0.9854

**Table 2:** Quantitative results for evaluation metrics on the “Bright cell” image with four other methods.

	QMI	QNCIE	QG	QP	QCB	PSNR	SSIM
DWT	1.8902	0.9074	0.9630	0.9547	0.9629	43.7463	0.9860
Quad-tree	1.8083	0.9007	0.9593	0.9372	0.9467	42.8896	0.9856
GFDF	1.5250	0.8818	0.8919	0.8816	0.5276	43.3569	**0.9874**
Ours	**1.** **9182**	**0.** **9098**	**0.96** **52**	**0.9** **578**	**0.9815**	**44.3206**	0.9870

**Table 3:** Blind image quality evaluation results for first dataset of conjunctival goblet cell images.

	DWT	Quad-Tree	GFDF	Ours
BRISQUE	42.590	43.459	35.619	**35.890**
NIQE	12.969	12.515	3.442	**2.955**

**Table 4:** Blind image quality evaluation results for second dataset of conjunctival goblet cell images.

	DWT	Quad-Tree	GFDF	Ours
BRISQUE	42.239	43.443	36.941	**31.006**
NIQE	11.768	12.063	3.883	**2.855**

**Table 5:** Blind image quality evaluation results for third dataset of conjunctival goblet cell images.

	DWT	Quad-Tree	GFDF	Ours
BRISQUE	42.668	43.499	**19.908**	31.676
NIQE	15.775	19.467	4.221	**3.28**
